# Determinants of unmet need for family planning in rural Burkina Faso: a multilevel logistic regression analysis

**DOI:** 10.1186/s12884-017-1614-z

**Published:** 2017-12-19

**Authors:** Joseph K. Wulifan, Albrecht Jahn, Hervé Hien, Patrick Christian Ilboudo, Nicolas Meda, Paul Jacob Robyn, T. Saidou Hamadou, Ousmane Haidara, Manuela De Allegri

**Affiliations:** 1 Faculty of Medicine, Institute of Public Health, University of Heidelberg, Heidelberg, Germany; 2 School of Business & Law, University for Development Studies, Wa, Ghana; 3 Centre Muraz, Avenue Mamadou Konate, Bobo-Dioulasso, Burkina Faso; 4 World Bank, 179 Avenue du President Saye Zerbo, Ouagadougou, Burkina Faso

**Keywords:** Unmet need, Unintended pregnancy, Family planning, Rural districts, Burkina Faso

## Abstract

**Background:**

Unmet need for family planning has implications for women and their families, such as unsafe abortion, physical abuse, and poor maternal health. Contraceptive knowledge has increased across low-income settings, yet unmet need remains high with little information on the factors explaining it. This study assessed factors associated with unmet need among pregnant women in rural Burkina Faso.

**Method:**

We collected data on pregnant women through a population-based survey conducted in 24 rural districts between October 2013 and March 2014. Multivariate multilevel logistic regression was used to assess the association between unmet need for family planning and a selection of relevant demand- and supply-side factors.

**Results:**

Of the 1309 pregnant women covered in the survey, 239 (18.26%) reported experiencing unmet need for family planning. Pregnant women with more than three living children [OR = 1.80; 95% CI (1.11–2.91)], those with a child younger than 1 year [OR = 1.75; 95% CI (1.04–2.97)], pregnant women whose partners disapproves contraceptive use [OR = 1.51; 95% CI (1.03–2.21)] and women who desired fewer children compared to their partners preferred number of children [OR = 1.907; 95% CI (1.361–2.672)] were significantly more likely to experience unmet need for family planning, while health staff training in family planning logistics management (OR = 0.46; 95% CI (0.24–0.73)] was associated with a lower probability of experiencing unmet need for family planning.

**Conclusion:**

Findings suggest the need to strengthen family planning interventions in Burkina Faso to ensure greater uptake of contraceptive use and thus reduce unmet need for family planning.

## Background

Many women in low and middle-income countries (LMICs) would like to limit or delay getting pregnant, but do not have access to consistent use of modern contraceptive methods [1, 2]. These women experience an unsatisfied demand for contraception, which is commonly referred to as *unmet need for family planning*. Unmet need for family planning (FP) is further defined as: *unmet need for limiting*, i.e. when the woman does not wish to have any more children; and *unmet need for spacing*, i.e. when the woman would have wished to delay the birth of her next child by at least 2 years [3–5].

The concept of unmet need for family planning is central to reproductive health policies, as it bears serious implications for the woman, the child, family and the society as a whole [6, 7]. In many LMICs, unmet need for family planning among pregnant women (unintended pregnancies i.e. pregnancies which are mistimed or unwanted at the time of conception) is a main cause of closely spaced births, childbearing at a very early age, unsafe abortions or physical abuse, all of which are considered main contributors to high maternal and infant mortality [3, 6–8]. In addition, as unmet need for family planning is closely related to high female illiteracy, gender inequality and poverty, this unsatisfied demand for family planning not only negatively impacts women’s reproductive health, but also their ability to participate in economic and educational activities necessary to overcome the cycle of poverty and ill-health [9–11].

For these reasons, counteracting unmet need for family planning has become a key global health priority, which is addressed by both the Millennium Development Goals 4 (reduction of child mortality) and 5 (improve maternal health and achieve universal access to sexual/reproductive healthcare) [12–14] and by the proposed sustainable development goals (SDGs) 2030 (Goal 3.1 reduce maternal mortality and 3.7 ensure universal access to reproductive health services including family planning) [7, 15]. Still, counteracting unmet need for family planning poses a number of challenges since access to and use of family planning methods is influenced by a number of factors acting at the individual, family, community, and health service level [1, 16, 17].

In Burkina Faso, fertility rates remain high at 6.0 children per woman. Contraceptive use is still low, with only 16% of all reproductive age women reporting use of any modern method of family planning in 2013 [18, 19]. As a consequence, unmet need for family planning remains high, at an estimated 29% in 2013 [3, 19]. In 2008, an estimated 32% of all pregnancies in the country were unintended and a third of these pregnancies were resolved through induced abortion [3, 20]. Studies in Burkina Faso indicate that conditions to enable most women to prevent unintended pregnancies are limited [18] and the risk of maternal death related to unsafe abortion complications is almost four in 10 women [3, 20, 21].

Still, research has paid very limited attention to understanding factors influencing unmet need for family planning, with the only available study being focused on causes and consequences of unintended pregnancies and induced abortions [3, 8, 20, 21] rather than on unmet need itself. Though many studies assessed contraceptive use among women of reproductive age in sub-Saharan Africa [22–24], only two of those focused on Burkina Faso, and both were published over a decade ago [25, 26].

This study seeks to fill this knowledge gap by examining determinants of unmet need for family planning among pregnant women in rural Burkina Faso. Findings are expected to guide policy makers interested in designing policies and programs aimed at reducing unmet need in an attempt to ultimately reduce maternal mortality and improve women’s health.

## Methods

### Study setting

The study used data from the baseline round of a survey which included multiple tools in order to evaluate the impact of a performance-based financing (PBF) intervention on access to and quality of a wide range of healthcare services. Specifically, this study used data from both the household survey and from the healthcare workers’ survey embedded within the larger set of tools needed for the impact evaluation. Both surveys were applied in the twenty-four (24) districts distributed in six (6) regions of Burkina Faso (Boucle du Mouhoun, Centre-Est, Centre-Nord, Centre-Ouest, Nord and Sud-Ouest) where PBF was to be rolled out starting in April, 2014.

### Study design, sample size and data collection

Data were collected from October 2013 to March 2014. The household survey relied on a three-stage cluster sampling technique. First, clusters were defined to reflect the catchment areas of the 448 health facilities in the 24 districts. Second, one village was selected in each of the 448 catchment areas. Third, fifteen (15) households were selected in each village. Households were selected on the basis of whether there was a woman living in the household who was currently pregnant or who had been pregnant in the twenty-four months prior to the survey date. Households were selected using a random route approach [27] until the desired sample size was achieved in each village.

Within a household, information was collected on the overall household socio-demographic and economic profile as well as on individual illness patterns, health care seeking behaviour, and related expenditure (for both adults and children).

Specifically, given our focus on unmet need for family planning among pregnant women, we considered as the effective sample for this study only the 1309 currently pregnant women included in the household survey. Currently pregnant women were asked whether their current pregnancy was intended, or whether they would have rather preferred not to have any more children, or to postpone the current pregnancy by at least 2 years. This allowed us to compute unmet need for family planning, further differentiating between unmet need for limiting and unmet need for spacing.

The healthcare workers’ survey targeted the staff working at all 443 facilities included in the study. Specifically, at each facility, the aim was to interview at least three healthcare workers. Respondents were conveniently selected among the staff present in the facility on the day of the survey. Information was collected through means of a structured, close-ended questionnaire with several modules, covering healthcare worker’s roles and responsibilities, their work environment, their training with specific reference to family planning, and facility assessment on availability of family planning methods.

Data collection was carried out by trained interviewers recruited and supervised by the colleagues at Centre-MURAZ. Both the household and the healthcare workers’ surveys relied on digital data collection, using Personal Digital Assistants (PDAs/mini computers) with data being sent to a central server on a daily basis using mobile phone connection.

### Study variables and their measurement

Table 1 reports the complete list of variables included in our analysis, which were derived from the household and healthcare workers’ survey, as well as the expected direction of the estimated coefficient. Information from the two surveys was merged into one dataset (matched at the health facility level) to account for the fact that a mixture of demand-side (i.e. pertaining to women, their partners, and their households) and supply-side (i.e. pertaining to health system) factors is expected to influence unmet need for family planning [28]. The outcome was defined as a dichotomous variable, differentiating between pregnant women with unmet need for family planning (coded as 1) and pregnant women without such unmet need (coded as 0). According to available information from WHO and demographic and health surveys (DHS) unmet need is estimated from non-contraceptive users (pregnant women and non-pregnant who are fecund and desire to have a child in at least 2 years’ time) [3, 6, 7]. Given the non-availability of information on the non-pregnant women explained in the methodological considerations section and the fact that evidence suggest that women (pregnant and non-pregnant) have differentiated needs and should be targeted in their different sects when designing family planning intervention, our focus on unmet need was among the pregnant women category [3, 6, 8, 17]. A pregnant woman was defined as having unmet need if she indicated in the questionnaire that her pregnancy was either wanted later (mistimed pregnancy) or she did not want to be pregnant (unwanted pregnancy) but was not using any method of contraception before the pregnancy. Women with mistimed pregnancies were classified as having unmet need for spacing while those with unwanted pregnancies were classified as having unmet need for limiting. These two (2) categories are referred to as total pregnant women with unmet need for family planning also referred to as women with unintended pregnancy consistent with the WHO and World Bank definition of unmet need among the pregnant women category which is often used as proxy for unmet need [28, 29]. Pregnant women who indicated that their current pregnancy was desired did not experience unmet need for family planning (intended pregnancies) [29].

**Table 1:** Variables, their distribution in the study sample, and the expected coefficient sign (*n* = 1309)

Variables	Measurement	Number	Percent	Expected coefficient sign
Outcome: Unmet need	0 = no unmet need	1070	81.74	NA
	1 = unmet need	239	18.26	
	Unmet need for spacing	197	15.05	
	Unmet need for limiting	42	3.21	
Explanatory:				
Woman’s age	15–49 years (continuous)			–
Number of children:				
	0 = Fewer than 4 children	1125	85.94	
	1 = 4 and more children	184	14.06	–
Number of living sons:				
	0 = No living son	516	39.42	–
	1 = At least one living son	793	60.58	
Experienced death of a child:				
	0 = Did not experience the death of a child	1050	19.48	+
	1 = Experienced the death of a child	259	19.79	
Children less than 1 year:				
	0 = Has no children younger than 1 year	255	19.48	–
	1 = Has a child younger than 1 year	1054	80.52	
Marriage type:				
	0 = Monogamy	808	61.73	+
	1 = Polygamy	501	38.88	
Woman’s religion:				
	0 = Muslim	800	61.12	–
	1 = Christian	509	38.27	
Wealth index				
	1 = Poorest	233	17.04	+
	2 = Second quintile	277	21.16	
	3 = Middle quintile	271	20.70	
	4 = Fourth quintile	261	19.94	
	5 = Least poor	277	21.16	
Residence				
	0 = Urban	101	7.72	+
	1 = Rural	1208	92.28	
Woman’s approval of contraceptive use				
	0 = Approve	1022	78.07	–
	1 = Disapprove	287	21.93	
Partner’s approval of contraceptive use				
	0 = Approve	724	60.69	–
	1 = Disapprove	585	39.31	
Couple’s discussion on contraceptive use	0 = Never	798	60.96	+
	1 = At least once	511	39.04	
Desired number of children (woman vs man)	0 = Same	621	47.44	–
	1 = Other	688	52.56	
Distance to Health Facility				
	0 = Less than 5 km	663	50.65	–
	1 = 5 or more km	646	49.35	
Barrier contraceptives				
	0 = Not available	156	11.92	+
	1 = Available	1153	88.08	
Hormonal contraceptives				
	0 = Not available	142	10.85	+
	1 = Available	1167	89.15	
IUD contraceptives				
	0 = Not available	888	67.84	+
	1 = Available	421	32.16	
Health worker training on FP				
	0 = Not trained	859	65.62	+
	1 = Trained	450	34.38	
Health worker training on FP logistics				
	0 = Not trained	1131	86.40	+
	1 = Trained	178	13.60	

Most of the independent variables included in Table 1 are self-explanatory. Number of living children was categorized into two groups with the classification being consistent with prior studies [30, 31]. We looked at sons living as important in relation to unmet need for family planning. In most patriarchal societies, male children are required to maintain the family lineage and as such, women are expected to give birth to male children. In line with prior research [32], household socio-economic status was assessed by computing a wealth index based on a combination of housing infrastructure and ownership of mobile goods, using multiple correspondence analysis.

Four variables, defined in the literature as proximate variables, were included as measures of a woman and her partner’s attitude and decision making towards family planning. Proximate variables are intermediate variables that focus on attitude and decision making [1, 33, 34]. In our analysis, they were: woman’s approval of contraceptive use; partner’s approval of contraceptive use; couple discussion on family planning; and woman’s desire for fewer children in relation to partner. Their inclusion was motivated by the existence of prior evidence suggesting that partners’ involvement in family planning decisions is a key factor shaping women’s reproductive behaviour. Evidence indicates that most women positively adopt family planning methods when they perceive their partner’s approval of contraceptive use [28, 35].

A set of variables from the health facility assessment and from the healthcare provider survey was included to account for health system factors likely to influence unmet need for family planning. Distance to the referral health facility was assessed around the cut-off point of 5 km, in line with WHO guidelines on accessibility [36, 37]. We included a measure of the contraceptives available at each facility, distinguishing between barrier contraceptives, hormonal contraceptives, and IUD. We included two variables to assess healthcare providers’ training, one looking at general training in family planning and one looking more specifically at logistics (procurement and stocking) concerning family planning products.

### Analytical approach

Bivariate analysis was carried out to assess non-adjusted associations between the single variables and unmet need for family planning. For each of the independent explanatory variables included in our final analysis, we estimated the crude odd ratio using univariate logistic regression. We used multivariate multilevel logistic regression to identify significant associations between unmet need for family planning and the explanatory variables, while controlling for potential confounders. Specifically, we used the Stata command xtlogit [38, 39]. The application of multilevel (random-effect) modelling was used to account for the fact that women were clustered at the district level. Preliminary analysis had in fact detected important differences in unmet need for family planning across districts (Table 2). We purposely did not account for clustering at the household level, given that we recorded multiple women only in 37 households.

**Table 2:** Unmet need for family planning by region and district

REGION			DISTRICT		
	No unmet need	Unmet need		No unmet need	Unmet need
	*N (%)*	*N (%)*		*N (%)*	*N (%)*
	1070 (81.74%)	239 (18.26%)		1070 (81.74%)	239 (18.26%)
Boucle du Mouhoun	216 (82.76)	45 (17.24)	Boromo	30 (81.08)	7 (18.92)
			Nouna	155 (86.59)	24 (13.41)
			Solenzo	6 (50.00)	6 (50.00)
			Toma	25 (75.76)	8 (24.24)
Centre-Est	116 (85.29)	20 (14.71)	Manga	22 (78.57)	6 (21.43)
			Ouargaye	45 (91.84)	4 (8.16)
			Tenkodogo	38 (82.61)	8 (17.39)
			Zabre	11 (84.62)	2 (15.38)
Centre-Nord	287 (85.16)	50 (14.84)	Barsalgho	24 (88.89)	3 (11.11)
			Kaya	204 (85.36)	35 (14.64)
			Kongoussi	16 (94.12)	1 (5.88)
			Ziniare	43 (79.63)	11 (20.37)
Centre-Ouest	180 (77.92)	51 (22.08)	Koudougou	92 (71.88)	36 (28.13)
			Nanoro	19 (82.61)	4 (17.39)
			Reo	58 (90.63)	4 (17.39)
			Sapouy	11 (68.75)	5 (31.25)
Nord	234 (79.86)	59 (20.14)	Bousse	44 (81.52)	10 (18.52)
			Gourcy	84 (78.50)	23 (21.50)
			Ouahigouya	62 (76.54)	19 (23.46)
			Yako	44 (86.27)	7 (13.73)
Sud-Ouest	37 (72.55)	14 (27.45)	Batie	11 (68.75)	5 (31.25)
			Dano	1 (50.00)	1 (50.00)
			Diebougou	18 (78.26)	5 (21.74)
			Gaoua	7 (70.00)	3 (30.00)

## Results

### Descriptive analysis

Out of the 1309 currently pregnant women included in the household survey, 1070 (81.74%) had intended to get pregnant, while 239 (18.26%) had not intended to (i.e. unintended pregnancies). This value represents the measure of unmet need for family planning, further distinguished between unmet need for spacing 197 (15.05%) and unmet need for limiting 42 (3.21%).

The second set of results in Table 3 reports unadjusted estimates for the association between unmet need and selected explanatory variables. A positive association was detected between unmet need for family planning and number of living children [OR = 1.72; 95% CI (1.19–2.48)], having a child younger than 1 year [OR = 1.56; 95% CI (1.06–2.32)], woman’s disapproval of contraceptive use [OR = 1.94; 95% CI (1.42–2.65)], partner’s disapproval of contraceptive use [OR = 2.51; 95% CI (1.10–2.07)], couple discussion on contraceptive use [OR = 1.33; 95% CI (1.00–1.77)] and a woman’s desire for children compared to partner [OR = 2.22; 95% CI (1.65–2.99)]. A negative association was detected between unmet need for family planning and distance to health facility [OR = 0.75; 95% CI (0.56–0.99)] and health worker training in contraceptive logistic management [OR = 0.46; 95% CI (0.27–0.77)]. No significant association was detected between unmet need and socio-economic status. Table 2 reports important differences in the level of unmet need for FP across districts with the highest rates in Solenzo and Dano (50.00% each), Sapouy and Batié (31.25% each), Gaoua (30.00%), Koudougou (28.13%) and the lowest in Kongoussi (5.88%), Ouargaye (8.16%), Reo (9.38%) and Barsalgho (11.11%).

**Table 3:** Unadjusted and adjusted odds ratio for unmet need for family planning

Variables	Unadjusted estimates		Adjusted estimates	
	OR	CI	OR	CI
Woman’s age (continuous)	1.00		1.00	
	1.01	0.98–1.03	0.97	0.94–1.00
Number of Children:				
Less than 4 children	1.00		1.00	1.11–2.91
4+ children	1.72	1.19–2.48	1.80	
Number of living sons:				
No living son	1.00		1.00	
At least one living son	1.30	0.97–1.75	1.20	0.80–1.80
Experienced death of a child:				
Did not experience the death of a child	1.00		1.00	
Experienced the death of a child	0.86	0.60–1.24	0.88	0.59–1.31
Children less than 1 year:				
Has no children younger than 1 year	1.00		1.00	
Has a child younger than 1 year	1.56	1.06–2.32	1.75	1.04–2.97
Marriage type:				
Monogamy and others	1.00		1.00	
Polygamy	1.04	0.77–1.40	0.88	0.64–1.21
Woman’s religion:				
Muslim	1.00		1.00	
Christianity & others	1.18	0.89–1.57	1.29	1.00–1.76
Wealth index:				
Poorest	1.0		1.00	
Second quintile	1.34	0.84–2.15	1.36	0.83–2.22
Middle quintile	1.48	0.92–2.36	1.54	0.94–2.52
Fourth quintile	0.80	0.48–1.34	0.88	0.51–1.52
Least poor	1.56	0.98–2.49	1.72	1.04–2.85
Residence				
Urban	1.00		1.00	
Rural	1.03	0.60–1.75	1.01	0.56–1.81
Woman’s approval of contraceptive use				
Approve	1.00		1.00	
Disapprove	1.94	1.42–2.65	1.48	1.00–2.21
Partner’s approval of contraceptive use				
Approve	1.00		1.00	
Disapprove	1.51	1.10–2.07	1.51	1.03–2.10
Couple’s discussion on contraceptive use				
Never	1.00		1.00	
At least once	1.33	1.00–1.77	1.48	1.09–2.02
Desired number of chn (woman vs man)				
Same	1.00		1.00	
Others	2.22	1.65–2.99	1.90	1.36–2.67
Distance to Health Facility				
Less than 5 km	1.00		1.00	
5 + km	0.75	0.56–0.99	0.72	0.53–0.98
Barrier contraceptives				
Not available	1.00		1.00	
Available	1.19	0.75–1.87	0.66	0.25–1.73
Hormonal contraceptives				
Not available	1.00		1.00	
Available	1.24	0.77–2.00	2.55	0.93–6.93
UD contraceptives				
Not available	1.00		1.00	
Available	0.82	0.60–1.12	0.75	0.53–1.06
Health worker training on FP				
Not trained	1.00		1.00	
Trained	1.04	0.77–1.39	1.28	0.92–1.79
Health worker training on FP logistics				
Not trained	1.00		1.00	
Trained	0.46	0.27–0.77	0.43	0.24–0.73
*Random effects*				
rho	–	–	8.88e-08	6.17e-20 - 1
Variance	–	–	−15.04	−89.09 – 58.99
Standard deviation	–	–	0.00	4.51e-40 – 6.48e + 12
*Model diagnostics*				
Log-likelihood	–	–	−571.79	
Wald chi2(22); p > chi2	–	–	89.16; 0.00	
Likelihood-ratio test of the rho			8.88e-08; *p* > 1.00	
(Chibar2(01); p > =chibar2	–	–		
Observations	1309		1309	
Clusters (Districts)	24		24	

### Multivariate multilevel analysis

The second set of results in Table 3 reports adjusted estimates from the multilevel logistic regression model. The model confirmed a positive association between unmet need for family planning and having four or more children [OR = 1.80; 95% CI (1.1–2.99)], having a child younger than 1 year [OR = 1.75; 95% CI (1.04–2.97)], being Christian [OR = 1.29; 95% CI (1.00–1.76)], a woman’s disapproval of contraceptive use [OR = 1.48; 95% CI (1.00–2.21)], partner’s disapproval of contraceptive use [OR = 1.51; 95% CI (1.03–2.10)], couple discussion on contraceptive use [OR = 1.48; 95% CI (1.09–2.02)] and facing a mismatch in the number of desired children with one’s partner [OR = 1.90; 95% CI (1.36–2.67)]. No consistent pattern was identified between unmet need and socio-economic status, although a significant positive association was identified between unmet need and the least poor income quintile [OR = 1.72; 95% CI (1.04–2.85)].

The model detected a negative association between unmet need for family planning and health workers’ training on contraceptive logistics [OR = 0.46; 95% CI (0.24–0.73)] and distance to health facility [OR = 0.72; 95% CI (0.53–0.98)]. The model did not detect any variance attributable at the district level (rho = 0), providing an indication that the demand-side and supply-side factors included in the analysis were sufficient to account for the heterogeneity in unmet need originally observed across districts.

## Discussion

This study represents a unique contribution to the literature since it is one of the very first studies looking explicitly at determinants for unmet need for family planning among pregnant women rather than looking at determinants for unmet need in general (both pregnant and non-pregnant women)- [25, 26] in Burkina Faso specifically and in sub-Saharan Africa more generally. The ability to bring together information from the demand-side and from the supply-side to explore heterogeneity in unmet need further strengthens our analytical approach, since unlike most prior quantitative studies [28], it allows us to account for individual, household, and health system factors at once.

Our study detected the prevalence for unmet need for family planning to be lower than what is usually reported in the literature on Burkina Faso, where the rate of unmet need is estimated at just below 30% [3, 19, 40, 41]. Differences between our estimates and previously published estimates may be due to differences in the estimation method used (assessing unmet need among pregnant women versus assessing unmet need among non-pregnant women) [42]. In addition, we ought to acknowledge that our sample was largely rural, where the wish for children may be higher, leading to lower estimates of unmet need than in samples pooling rural and urban women together. Lastly, one cannot exclude that in the time elapsed between prior studies and our own, prevalence of contraceptive use increased, leading to a decrease in the prevalence of unmet need. Further population-based studies are therefore needed to confirm or refuse this hypothesis [3, 19].

Still, indicating that nearly one in five women in a consensual union experiences unmet need for family planning suggests that the problem remains of considerable magnitude and that action is urgently needed to fill the obvious gap in contraceptive use, currently estimated at a low 16% [19]. Women with unmet need are often the focus of family planning programs because they exhibit a discrepancy between their fertility intentions and contraceptive use [43, 44]. Still, our findings indicate that current efforts have not been sufficient to reach and ensure use among all women who would potentially wish to limit or delay pregnancies. Unmet need bears important consequences for a woman and her family, including unwanted pregnancies, unsafe abortions, and poor maternal health outcomes. Ultimately, overcoming unmet need among rural women is likely to result in significant improvements in women’s health while also limiting further population growth to enable the country to overcome some challenges towards sustainable development [3, 35].

Beyond providing a current estimate of unmet need based on population-based data, our study identified a number of relevant factors likely to shape the probability that a woman experiences unmet need. Attention can be drawn to the fact that the heterogeneity in unmet need observed across districts could largely be explained by the individual, household, and health system factors included in our analysis, as reflected by the insignificant value of the rho. This finding is suggestive of the fact that heterogeneity in unmet need is not driven by structural differences across districts, but rather by clustering of individual, household, and health system characteristics across these districts.

Before discussing in detail the role of individual and household factors, it is interesting to note how the regression model confirmed that health workers’ training in family planning logistics was a supply-side factor bearing a significant impact on unmet need for family planning. The most plausible explanation, however, is that the variable included in the analysis captured continuity in contraceptive supply at the facility level rather than mere availability of such products at the time of the interview. It is to be expected that health workers specifically trained in family planning logistics would be better managers of their supply and may even engage in outreach activities to distribute the needed products.

Pregnant women having four or more living children were found to be significantly associated with unmet need for family planning, although our hypothesis had postulated exactly the opposite, based on the assumption that contraceptive use would be higher among women with several children meaning these pregnant women retrospectively were not among contraceptive users hence the unplanned pregnancies and the consequent unmet need [31, 45–48]. While further qualitative research is needed to unravel the reasons behind this finding, we can already speculate that women with many children actually wish to prevent further pregnancies, but are not empowered to do so by the socio-cultural setting in which they live [30, 49, 50]. Specifically, we can relate this finding to ones indicating a significant association between unmet need and the proximate variables included in our analysis. In line with prior research [26, 51, 52], our study indicates that women may be hampered in controlling their fertility because they are unable to discuss the matter with their partners and/or their partner clearly disapproves of contraceptive use. While the bulk of the evidence has traditionally pointed at men being opposed to contraceptive use out of fear of losing their role as heads of the family or indirectly encouraging their spouses to be promiscuous [11, 53–55], recent studies have suggested that men are increasingly more prone to accept contraceptive use, although their wives do not know it since the matter is rarely discussed openly [28, 47]. Convincing men to support contraceptive use, bridging the communication gap between men and women, and involving partners in family planning decisions are likely to be the most salient factors to increase the uptake of contraceptives and thus reduce unmet need for family planning. In addition, one ought to consider the influence of the socio-cultural setting even beyond the preferences of the single women and their spouses. To meet societal expectations in communities where women’s status is closely related to childbearing, women may initiate pregnancies at a young age and may continue to have children, even though they would wish otherwise [9, 11, 53–55], and even though their partners would approve of contraceptive use [56–62].

Also contradicting our initial hypothesis, our findings revealed that pregnant women with a living child below 1 year of age were significantly more likely to experience unmet need for family planning. This finding is worrisome since it indicates that pregnancies are not properly spaced in spite of women’s wish to do so. Clinical evidence indicates that the childbirth interval should be at least 2 years [4, 42], since closely spaced births have consistently been found to be associated with an increase in morbidity and mortality for both mothers and their babies [42, 63]. High levels of unmet need among women with infants are probably an indication of failures in postpartum family planning counselling and call into question the comprehensiveness and quality of post-natal care. It is also plausible to assume that high levels of unmet need among women with infants result from the false belief that women cannot conceive while breastfeeding [64, 65]. Although not unique to our setting [63–67], these findings indicate the urgent need to reform the content and quality of the information shared with women and their partners in the post-partum period to allow for informed family planning decision-making.

Further qualitative research is needed to explain the association we observed between unmet need and both a woman’s socio-economic status and distance to the health facility. Rather counterintuitively, our findings revealed that the least poor women and those living closer to the healthcare facility were the ones most likely to experience unmet need. At this stage, we can only speculate as to why unmet need appears to be lowest among the most vulnerable, i.e. poorer women and those living far from a facility. One plausible explanation may be tied to the fact that family planning is not always managed at the facility level, since relevant counselling and service provision is provided as a component of outreach activities, potentially addressing specifically the most vulnerable women. While a pro-vulnerable approach may be desirable per se, our findings point to the urgent need to reform service delivery since family planning efforts are expected to enable all women to make informed choices about their reproductive life.

### Methodological considerations

As we appraise the generalizability of our findings, we need to acknowledge the possibility that the associations observed between unmet need and the selected explanatory variables may be context-dependent and not necessarily applicable to other LMICs, even within SSA. Moreover, we need to report that the research team in selecting the explanatory variables to be included in the analysis, there was the possibility that additional explanatory variables not available in our survey may also be relevant in shaping unmet need. Similarly, we ought to recognize that the analysis relied exclusively on quantitative methods, thus making it impossible for the research team to investigate the role of unmeasurable dimensions, such as cultural beliefs and values. We must also mention the estimation of unmet need which used a population of currently pregnant women only. It would have been desirable to replicate the analysis on the entire sample of non-pregnant women of reproductive age included in our survey, but this was not possible due to the unavailability of relevant data to estimate unmet need for the non-pregnant fertile women. Last, but not least, we need to acknowledge the limitation of our quantitative study in addressing an issue as sensitive as family planning. We recognize that qualitative methods of inquiry may be better suited to explore reasons behind low adoption of contraceptives in low-income settings. Nevertheless, we trust that, by identifying a few key associations, quantitative research can be instrumental in generating impetus and working hypotheses for further qualitative inquiry.

## Conclusion

With almost one in five women experiencing unmet need for family planning, our study detects some of the highest rates reported in low income settings with socio-demographic characteristics similar to those of Burkina Faso [68]. Our findings further indicate a need to expand family planning efforts, targeting specifically women with several children and women in the immediate post-partum period as well as their partners, to fill the knowledge and communication gap to which our findings appear to point. Community-based family planning interventions should be considered as an accompanying measure to strengthen current service provision and reach a larger number of women [50, 69]. Enhancing women’s access to family planning will be instrumental in counteracting the current trend for unmet need in Burkina Faso.

## Abbreviations

FP: Family planning;
LMIC: Low and middle income country

## References

[1] Bongaarts J, Bruce J (1995). The causes of unmet need for contraception and the social content of services. Stud Fam Plan.

[2] Westoff CF (1992). Measuring the unmet need for contraception. Comment on Bongaarts. Popul Dev Rev.

[3] Bankole A, Hussain R, Sedgh G, Rossier C, Kaboré I, Guiella G (2014). Unintended pregnancy and induced abortion. Burkina Faso: causes and consequences.

[4] Nortman DL (1982). Measuring the unmet need for contraception to space and limit births. Int Fam Plan Perspect.

[5] Westoff CF, Pebley AR (1981). Alternative measures of unmet need for family planning in developing countries.

[6] Okonofua FE, Odimegwu C, Ajabor H, Daru PH, Johnson A (1999). Assessing the prevalence and determinants of unwanted pregnancy and induced abortion in Nigeria. Stud Fam Plan.

[7] USAID (2009). Family planning and the MDGs. Saving lives, saving Ressources. Task Order.

[8] Sengh G, Bankole A, Singh S. Women with an unmet need for contraception in developing countries and their reasons for not using a method. 2007 p. 80. Report No.: 37.

[9] Kabagenyi A, Jennings L, Reid A, Nalwadda G, Ntozi J, Atuyambe L (2014). Barriers to male involvement in contraceptive uptake and reproductive health services. A Qualitative Study of Men and Women’s Perceptions in Two Rural Districts in Uganda. Reprod Health.

[10] Longwe A, Smits J, de Jong E. Number and spacing of children and women’s employment in Africa. 2013 [cited 2014 May 20]; Available from: http://www.ru.nl/publish/pages/516298/nice-13103.pdf

[11] Mosha I, Ruben R, Kakoko D (2013). Family planning decisions, perceptions and gender dynamics among couples in Mwanza, Tanzania. A Qualitative Study. BMC Pub Health.

[12] Cates WJ (2010). Family planning. The essential link to achieving all eight millennium development goals. Contraception.

[13] Hill K, Choi Y. Maternal mortality in 2000. Estimates developed by WHO, UNICEF and UNFPA. Geneva: World Health Organization; 2004.

[14] WHO (1999). HRP - UNDP/UNFPA/World Bank special Programme of research, development and research training in human reproduction. Kenyan men interested in family planning, but can the available services address their needs?. Soc Sci Res Policy Briefs.

[15] Horayangkura P. Open working group proposal for sustainable development goals. Document A/68/970. 2015;5:2–24. Available at http://undocs.org/A/68/970.

[16] Blanc AK, Way AA (1998). Sexual behavior and contraceptive knowledge and use among adolescents in developing countries. Stud Fam Plan.

[17] Casterline JB, Sinding SW (2000). Unmet need for family planning in developing countries and implications for population policy. Popul Dev Rev.

[18] Central Intelligence Agency, USA. The world Factbook. Africa, Burkina Faso; 2014.

[19] Population Reference Bureau (2014). Population and economic development 2014 data sheet.

[20] Sedgh G, Rossier C, Kaboré I, Bankole A, Mikulich M (2011). Estimating abortion incidence in Burkina Faso using two methodologies. Stud Fam Plan.

[21] Bankole A, Singh S, Hass T (1998). Reasons why women have induced abortions: evidence from 27 countries. Int Fam Plan Perspect.

[22] Adanu RM, Seffah J, Anarfi JK, Lince N, Blanchard K (2012). Sexual and reproductive health in Accra, Ghana. Ghana Med J.

[23] Okech TC, Wawire NW, Mburu TK (2011). Contraceptive use among women of reproductive age in Kenya’s City slums. Int J Bus Soc Sci.

[24] Palamuleni ME (2013). Socio-economic and demographic factors affecting contraceptive use in Malawi. Afr J Reprod Health.

[25] Babalola S, Vonrasek C (2005). Communication, ideation and contraceptive use in Burkina Faso: an application of the propensity score matching method. J Fam Plann Reprod Health Care.

[26] Sando B, Sya D, Pare R, Kouanda S, Savadogo L. The use of contraceptive methods by the Mossi in a rural health district of Kaya. Burkina Faso: Montrouge; 2001. 11440887

[27] Ayah R, Joshi MD, Wanjiru R, Njau EK, Otieno CF, Njeru EK (2013). A population-based survey of prevalence of diabetes and correlates in an urban slum community in Nairobi, Kenya. BMC Public Health.

[28] Korra A. Attitudes toward family planning and reasons for nonuse among women with unmet need for family planning in Ethiopia. Calverton: ORC Macro Calverton; 2002.

[29] Westoff CF (2006). New estimates for unmet need and demand for family planning.

[30] Imasiku ENS, Odimegwu CO, Adedini SA, Ononokpono DN (2014). Variations in unmet need for contraception in Zambia. Does ethnicity play a role?. J Biosoc Sci.

[31] Omane-Adjepong M, Oduro FT, Annin K (2012). A multinomial regression analysis of unplanned pregnancies in Ahafo Ano South District, Ghana. Am Int J Contemp Res.

[32] Vyas S, Kumaranayake L (2006). Constructing socio-economic status indices: how to use principal components analysis. Health Policy Plan.

[33] Westoff CF, Bankole A (2000). Trends in the demand for family limitation in developing countries. Int Fam Plan Perspect.

[34] Woldemicael G, Beaujot R (2011). Currently married women with an unmet need for contraception in Eritrea. Profile and determinants. Can Stud Popul.

[35] Adebowale SA, Palamuleni ME (2014). Determinants of unmet need for modern contraception and reasons for non-use among married women in rural areas of Burkina Faso. Afr Popul Stud.

[36] Jones S, Wardlaw J, Crouch S, Carolan M (2011). Modelling catchment areas for secondary care providers: a case study. Health Care Manag Sci.

[37] Diesfeld HJ (1973). The definition of the hospital catchment area and its population as a denominator for the evaluation of hospital returns in developing countries. Int J Epidemiol.

[38] Acock AC (2014). A gentle introduction to Stata.

[39] Long JS, Freese J (2001). Regression models for categorical dependent variables using Stata.

[40] Gribble J (2008). Family planning in Ghana, Burkina Faso, and Mali [internet].

[41] World Bank. Contraceptive prevalence in Burkina Faso. World Bank- Ctry. Website-2015; 2010.

[42] Bradley SE, Croft TN, Fishel JD, Westoff CF (2012). Revising unmet need for family planning- DHS analytical studies 25.

[43] Bongaarts J. The KAP-gap and the unmet need for contraception. Popul Dev Rev. 1991;17(2):293–313.

[44] Westoff CF. Is the KAP-gap real? Popul Dev Rev. 1988;14(2):225–32.

[45] Hamdela B, G/mariam A, Tilahun T (2012). Unwanted pregnancy and associated factors among pregnant married women in hosanna town, southern Ethiopia. PLoS One.

[46] Ikamari L, Izugbara C, Ochako R (2013). Prevalence and determinants of unintended pregnancy among women in Nairobi, Kenya. BMC Pregnancy Childbirth.

[47] Palamuleni ME, Adebowale A (2014). Prevalence and determinants of unintended pregnancies in Malawi. Afr Popul Stud.

[48] Tebekaw Y, Aemro B, Teller C (2014). Prevalence and determinants of unintended childbirth in Ethiopia. BMC Pregnancy Childbirth.

[49] Hailemariam A, Haddis F (2011). Factors affecting unmet need for family planning in southern nations, nationalities and peoples region, Ethiopia. Ethiop. J Health Sci.

[50] Cleland J, Harbison S, Shah IH (2014). Unmet need for contraception: issues and challenges. Stud Fam Plan.

[51] Lasee A, Becker S (1997). Husband-wife communication about family planning and contraceptive use in Kenya. Int Fam Plan Perspect.

[52] Mahmood N (1998). Reproductive goals and family planning attitudes in Pakistan: a couple-level analysis. Pak Dev Rev.

[53] Bawah AA, Akweongo P, Simmons R, Phillips JF (1999). Women’s fears and Men’s anxieties. The impact of family planning on gender relations in northern Ghana. Stud. Fam. Plann.

[54] Hall MAK, Stephenson RB, Juvekar S (2008). Social and logistical barriers to the use of reversible contraception among women in a rural Indian Village. J Health Popul Nutr.

[55] Kaida A, Kippi W, Hessel P, Konde-Lule J (2005). Male participation in family planning. Results from a qualitative study in Mpigi District,Uganda. J Biosoc Sci.

[56] Abeykoon A (1995). Sex preference in south Asia: Sri Lanka an outlier. Asia Pac Popul J.

[57] Arnold F, Sathar ZA, Phillips JF (1996). Son preference in South Asia. Fertility transition in South Asia.

[58] Arnold F (1997). Gender preferences for children. U. S. agency Int. dev. USAID- off. Popul. Reprod. Health bur. Glob. Health.

[59] Bairagi R (2001). Effects of sex preference on contraceptive use, abortion and fertility in Matlab, Bangladesh. Int Fam Plan Perspect.

[60] Clark S (2000). Son preference and sex composition of children: evidence from India. Demography.

[61] Edlund L (1999). Son preference, sex ratios, and marriage patterns. J Polit Econ.

[62] Das Gupta M, Zhenghua J, Bohua L, Zhenming X, Chung W, Hwa-Ok B (2003). Why is son preference so persistent in east and South Asia? A cross-country study of China, India and the Republic of Korea. J Dev Stud.

[63] Winikoff B (1983). The effects of birth spacing on child and maternal health. Stud Fam Plan.

[64] Segal SJ, Tietze SL, Lincoln R (1987). The effect of breastfeeding on the rate of conception. Fertil. Regul. Public health.

[65] Labbok MH, Perez A, Valdes V, Sevilla F, Wade K, Laukaran VH (1994). The Lactational amenorrhea method (LAM): a postpartum introductory family planning method with policy and program implications. Adv Contracept.

[66] WHO. Ensuring human rights in the provision of contraceptive information and services. Geneva: WHO; 2014. 24696891

[67] Ross JA, Winfrey WL (2001). Contraceptive use, intention to use and unmet need during the extended postpartum period. Int Fam Plan Perspect.

[68] Singh S, Sedgh G, Hussain R (2010). Unintended pregnancy: worldwide levels, trends, and outcomes. Stud Fam Plan.

[69] Kumpfer KL, Alvarado R, Smith P, Bellamy N (2002). Cultural sensitivity and adaptation in family-based prevention interventions. Prev Sci.

